# In Vivo Whole-Cell Patch-Clamp Methods: Recent Technical Progress and Future Perspectives

**DOI:** 10.3390/s21041448

**Published:** 2021-02-19

**Authors:** Asako Noguchi, Yuji Ikegaya, Nobuyoshi Matsumoto

**Affiliations:** 1 Graduate School of Pharmaceutical Sciences, The University of Tokyo, Tokyo 113-0033, Japan; asakonoguchi.an@gmail.com (A.N.); yuji@ikegaya.jp (Y.I.); 2 Institute for AI and Beyond, The University of Tokyo, Tokyo 113-0033, Japan; 3 Center for Information and Neural Networks, National Institute of Information and Communications Technology, Suita City, Osaka 565-0871, Japan

**Keywords:** patch-clamp recording, whole-cell recording, rat, mouse, membrane potential, local field potential, hippocampus, neocortex, axon, dendrite

## Abstract

Brain functions are fundamental for the survival of organisms, and they are supported by neural circuits consisting of a variety of neurons. To investigate the function of neurons at the single-cell level, researchers often use whole-cell patch-clamp recording techniques. These techniques enable us to record membrane potentials (including action potentials) of individual neurons of not only anesthetized but also actively behaving animals. This whole-cell recording method enables us to reveal how neuronal activities support brain function at the single-cell level. In this review, we introduce previous studies using in vivo patch-clamp recording techniques and recent findings primarily regarding neuronal activities in the hippocampus for behavioral function. We further discuss how we can bridge the gap between electrophysiology and biochemistry.

## 1. Introduction

**Figure 1:**
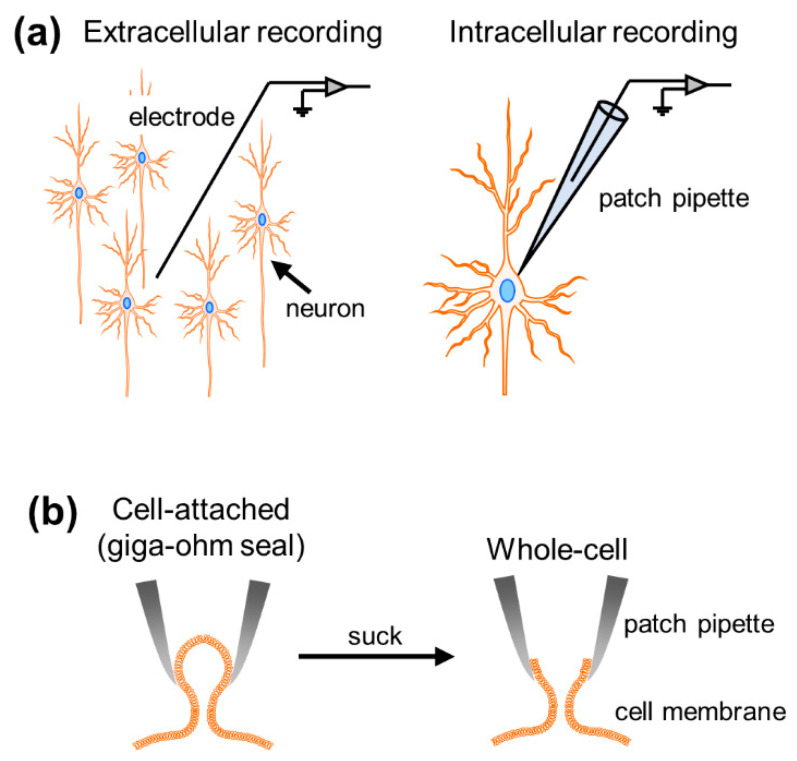
Overview of the patch-clamp method. (**a**) Comparison between extracellular and intracellular recordings. The patch-clamp method is included in the intracellular recording. (**b**) The patch-clamp method has at least two configurations. The tip of the patch pipette is tightly attached to the cell membrane, and the firing activity of a single neuron is recorded; this is called the cell-attached configuration (*left*). Then, the membrane is sucked, and the transmembrane current through the whole cell is recorded; this is called the whole-cell configuration (*right*). Some schematic drawings in Figures 1–5 are generated by modifying images available from Motifolio illustration toolkits (Motifolio Inc., Ellicott City, MD, USA).

Well-orchestrated multineuronal action potentials, each of which is triggered by spatiotemporal summation of synaptic inputs, support brain functions. To investigate neural activity, researchers have developed various types of electrophysiological recording techniques, roughly divided into extracellular and intracellular methods. While extracellular recordings enable us to obtain data on neuronal firing and collective oscillatory dynamics generated by multiple cells surrounding recording electrodes, intracellular recordings allow for the measurement of subthreshold membrane potential dynamics and suprathreshold firing activity at the single-cell level (Figure 1a). These intracellular recordings are further classified into patch-clamp and sharp electrode techniques in terms of the thickness of the recording electrode tip. In particular, the higher impedance of sharp electrodes results in large leak currents and precludes voltage-clamp recording. The patch-clamp method is the only way to capture the intracellular activity of single neurons with a high signal-to-noise ratio [1].

When researchers attempt to patch-clamp neurons, they first heat and pull glass capillaries to obtain thin glass pipettes with small tips (diameter of ~3 µm and resistance of 3–7 MΩ) [2]. They then fill the pipette with artificial intracellular solution and attach the pipette with a holder in a recording device so that an AgCl-coated wire (protruding from the holder) can be inserted. After completing the preparation, they press the recording pipette onto the cell membrane and tightly seal the membrane with a resistance of >1 GΩ between the pipette and the membrane, named the giga-ohm seal. This configuration is also called the “cell-attached mode” (Figure 1b). This recording mode enables us to capture the dynamics of the membrane currents generated by ions through ion channels on the cell membrane. Historically, the patch-clamp method was originally developed to examine single-channel electrical currents [1]. Once negative pressure is applied and a small hole is made on the cell membrane (“whole-cell mode”), the net dynamics of somatic currents and voltages generated through all ion channels expressed on the cell membrane can be measured. Somatic currents, reflecting synaptic inputs to the neuron, are recorded by holding voltage at a specific value (“voltage-clamp”; [3]), whereas not only action potentials but also membrane potential fluctuations below the firing threshold can be recorded by the clamping current (“current-clamp”). Although there are other recording modes in the patch-clamp method [4], we will focus on the whole-cell recording mode in both voltage- and current-clamp configurations.

In this review, we provide a general description of patch-clamp techniques and simultaneously look back over past findings using in vivo whole-cell recordings from anesthetized or awake animals in Section 2 and Section 3 [5,6]. In Section 2 and Section 3, we introduce novel findings that could be demonstrated only by in vivo whole-cell recordings, focusing primarily on the hippocampus. We then introduce recent methodological advances from the viewpoint of combination with other techniques and discuss future perspectives of this longstanding technique in Section 4 and Section 5.

## 2. In Vivo Whole-Cell Recordings from Anesthetized Animals

### 2.1. Neocortex

In vivo whole-cell recordings were first obtained from anesthetized animals [7]. This is because establishing a stable giga-ohm seal is a critical process of whole-cell recordings with a high signal-to-noise ratio requiring the brain to be as stationary as possible. Targeted regions in an early stage were the primary visual cortex (V1) [5,7] and the primary somatosensory cortex (S1) [8,9,10,11,12], as these cortices are near the surface and are relatively more accessible than other neocortical areas [13,14,15]. Indeed, our group previously succeeded in double in vivo whole-cell patch-clamp recordings from the mouse neocortex [16]. Researchers patched neurons (to be recorded) without actual visual monitoring of neuronal morphology and electrode location, which is called a “blind patch-clamp” [17].

**Figure 2:**
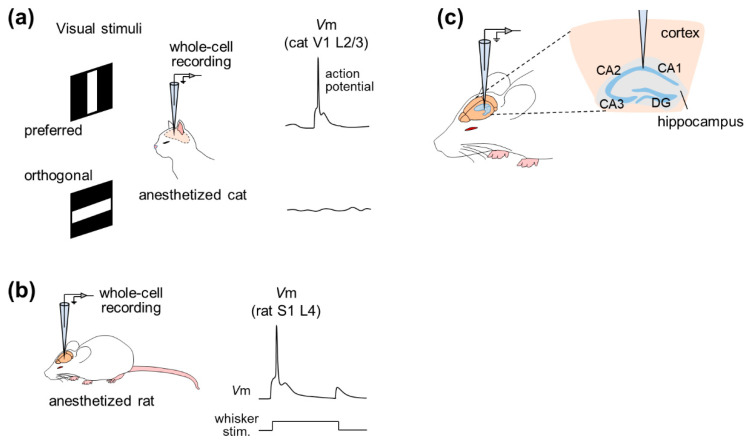
Examples of studies based on in vivo whole-cell recordings from anesthetized animals. (**a**) Whole-cell recordings are made from neurons in layer 2/3 of the primary visual cortex (V1) of an anesthetized cat while the animal is exposed to visual stimuli. In this example, the membrane potentials are depolarized when the cat sees the preferred (horizontal) stimuli, whereas the potentials remain stable when the animal is exposed to the orthogonal (vertical) stimuli. (**b**) Whole-cell recordings are made from neurons in layer 4 of the primary somatosensory cortex (S1) of an anesthetized rat. When the rat was given whisker stimuli, the membrane potentials of the patched neuron were depolarized. (**c**) Whole-cell recordings are made from neurons in the hippocampus beneath the neocortex. *Abbreviations*: V1, primary visual cortex; S1, primary somatosensory cortex; DG, dentate gyrus.

In 1994, whole-cell recording was used to examine the effect of GABAergic inputs on the orientation selectivity of cat V1 pyramidal neurons under anesthesia, focusing on supra- and subthreshold activity [7] (Figure 2a). This study revealed that even though subthreshold activity was changed after intracellular application of GABA receptor blockers through a patch pipette, V1 neurons still fired action potentials in an orientation-selective manner, suggesting that excitatory inputs were sufficient to generate orientation selectivity.

Subsequently, the whole-cell recording method was applied, especially to the rat barrel cortex, a subregion of S1 [18], whose activity is correlated with whisker tactile behavior [8,9,10,11]. Intrinsic properties and subthreshold responses to whisker (mystacial vibrissae) stimulation were recorded by the whole-cell configuration under pentobarbital anesthesia [8]. A series of studies then precisely described dynamic receptive fields for whisker deflections by recordings from the rat barrel cortex under urethane anesthesia and *post hoc* reconstruction of recorded cells [9,10,11] (Figure 2b). In addition to post hoc visualization or reconstruction, patch-clamp recording was also combined with genetic manipulation and optical imaging of cortical neurons of urethane-anesthetized rats [19]. This study used lentiviral vectors for neuron-specific gene delivery to analyze the phenotype at the single cortical cell level using two-photon microscopy-based techniques. This study used high-resolution two-photon time-lapse imaging to monitor the structures of dendritic spines and axons and simultaneously measured cellular responses electrophysiologically by two-photon microscopy-guided whole-cell recordings. This approach is ideally useful for associating electrophysiological function with gene expression in individual neurons in the intact brain, but it is still awaiting feasibility in awake animals.

### 2.2. Hippocampus and Other Regions

While these studies focused on the neocortex in the cerebrum, whole-cell recordings from anesthetized animals have investigated other regions, such as (i) the cerebrum (including the entorhinal cortex [20,21], the hippocampus [22,23,24,25,26,27,28,29,30,31], the basolateral amygdala [32,33,34], the piriform cortex [35,36,37], and the thalamus [38]) and even (ii) the brainstem (including the midbrain [39,40] and the pons [41]) and (iii) the cerebellum [42,43,44,45,46,47,48].

Hahn et al. first achieved in vivo whole-cell recordings from entorhinal pyramidal cells, hippocampal pyramidal cells, dentate granule cells and even hippocampal interneurons of unconscious animals [20,23,24] (Figure 2c). Simultaneous recordings of the neocortical local field potentials (LFPs) and membrane potentials of CA1 pyramidal cells, CA3 pyramidal cells and dentate granule cells under urethane anesthesia revealed that these three cell types were differentially modulated by cortical network oscillations, indicating differential functional connectivity between the neocortex and the hippocampal subfields [23]. Furthermore, the membrane potentials of hippocampal interneurons located at the border of the *stratum radiatum* and the *stratum lacunosum-moleculare* were phase-locked to neocortical phasic (also known as up–down) states with a small delay, suggesting that neocortical activity drives hippocampal interneurons during up–down states [24]. Recently, dual whole-cell recordings have been used to investigate the relationships between hippocampal neurons [31].

Whole-cell recordings of neurons in the basolateral amygdala (BLA), located much deeper than the hippocampus in vivo, have all been conducted under urethane anesthesia [32,33,34]. The studies showed that BLA neurons displayed slow oscillations emerging at a frequency of approximately 0.3 Hz. Using somatosensory stimuli (i.e., footshocks), auditory stimuli or posterior thalamus stimulation during up or down states, the studies suggested that oscillatory activity in the BLA was driven by ensembles of cortical neurons and that these ensembles gated the responses of amygdala neurons to aversive stimulation in a state-dependent manner; that is, aversive stimulation was effective when the network was in the down state but ineffective when the network was in the up state [32,34].

Brecht and Sakmann achieved in vivo whole-cell recordings from thalamic neurons in 2002 [38]. As the ventral posterior medial nucleus (VPM) of the thalamus is the major source of whisker-driven input to the barrel cortex, they targeted this brain area and described two main classes of VPM neurons: single-whisker excitation cells and multiwhisker excitation cells. The former showed sub- or suprathreshold responses to stimulation of a specific single whisker, whereas the latter exhibited responses to stimulation of multiple whiskers. Moreover, they demonstrated that these two cell types were different in the sizes of receptive fields, responding patterns to whisker deflection, the strength of inhibitory inputs, and the intrinsic properties [38].

A series of studies by Häusser’s group (including Chadderton et al., Rancz et al., Duguid et al., and Ishikawa et al.) investigated information processing in the cerebellum [42,43,44,45,46]. The cerebellum is a favorable model system for addressing the relationships between sensory-evoked synaptic inputs and the resulting pattern of output spikes because granule cells in the cerebellum constitute the input layer, translating mossy fiber signals into parallel fiber input to Purkinje cells. For example, Ishikawa et al. addressed the question of how multisensory (i.e., somatosensory, auditory, and visual) signals are integrated by single cerebellar granule cells at the input stage of the cerebellar cortex [45]. Using whole-cell voltage-clamp recordings, they described neurons responding to sensory, auditory, visual stimulation or the convergence of these stimulations and showed that the combination of multisensory inputs can enhance granule cell spike outputs.

In contrast to blind patch-clamp techniques, the “targeted patch-clamp” technique was developed by researchers to record membrane potentials from specific target cells in the neocortex. This method consists of “two-photon targeted patching” [49,50] and “shadow patching” [51,52,53,54].

**Figure 3:**
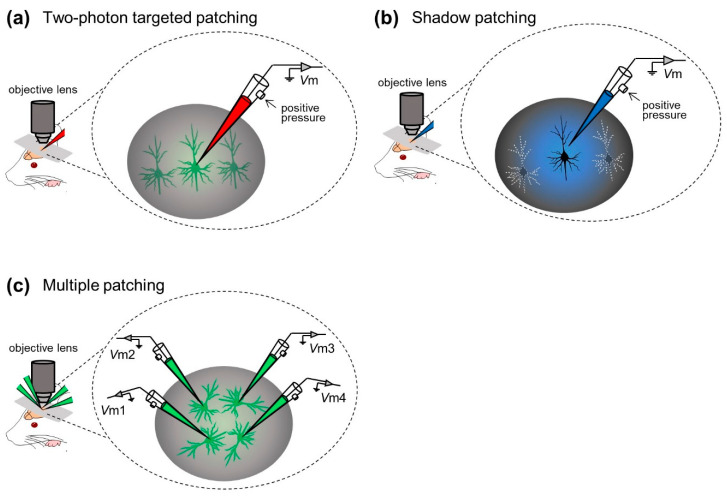
Examples of visually guided in vivo whole-cell recording techniques. (**a**) Whole-cell recordings are made from neocortical neurons with the aid of two-photon microscopy. (**b**) Whole-cell recordings are made from neocortical neurons under visual monitoring of the shadow of neuronal morphology. (**c**) Whole-cell recordings are made simultaneously from up to four neurons. *Abbreviation*: *V*m, membrane potential.

Margrie et al. first incorporated two-photon imaging into the in vivo patch-clamp method and developed in vivo targeted patching techniques to guide patch pipettes to individual, genetically labeled cortical neurons in vivo [49] (“two-photon targeted patching”; Figure 3a). Using genetically manipulated mice whose parvalbumin-positive interneurons were tagged with enhanced green fluorescent protein (eGFP), Margrie et al. made recordings from parvalbumin-positive interneurons in S1. They described the intrinsic properties of the interneurons and thereby revealed spontaneous and sensory-evoked activity patterns of S1 neurons [49]. Additionally, Kitamura et al. established the shadow patching method, where a patch electrode was used to perfuse the extracellular space of the targeted neuron with a fluorescent dye, enabling visualization of the neuron as a negative image (“shadow”) and identification based on its somatic and dendritic structures. They then placed the same electrode on the neuron under visual control to obtain patch-clamp recordings from visually identified neurons in the neocortex and cerebellum of rats and mice (“shadow patching”; Figure 3b). They also utilized targeted in vivo single-cell electroporation of plasmid DNA into identified cell types, leading to stable transgene expression [51]. These techniques have accelerated not only electrophysiological recording but also labeling and genetic manipulation of single neurons of intact naïve animals.

In most studies above, researchers captured membrane potential dynamics from a single neuron in a trial. Especially under the “blind” condition, even a single whole-cell configuration can only be achieved probabilistically (but see [55]). In general, the simultaneous whole-cell configuration of multiple neurons is technically challenging because movements of multiple pipettes interfere with each other and disrupt stable sealings; however, Jouhanneau et al. approached this issue by targeted patch-clamp recording simultaneously from up to four neocortical neurons [56,57,58,59] (Figure 3c). They described similarities or differences in information processing between recorded cells at the subthreshold level [56] and revealed synaptic connectivity between cortical neurons [57,59].

## 3. In Vivo Whole-Cell Recordings from Awake Animals

### 3.1. Neocortex

Margrie et al. trained rats to be almost immobile in a recording apparatus several days prior to surgery and recording. This familiarization is laborious but important because unhabituated animals often struggle or try to escape from the recording apparatus. The authors first reported whole-cell recordings from the barrel cortex of awake rodents and observed membrane potential depolarizations in response to whisker stimulation [6]. Subsequently, Poulet et al. and Yu et al. demonstrated that the desynchronized state in the whisker barrel cortex of mice during voluntary whisker behavior was triggered by increased firing activity of the thalamus [60] and correlated with the activity of a subset of interneurons [61].

Other groups, including us, have reported subthreshold responses to external stimuli in the V1 of conscious rodents [62,63,64,65,66,67,68] and behaving monkeys [69]. Furthermore, researchers applied in vivo whole-cell recordings to other cortical regions of awake rodents, including the primary motor cortex (M1) [70], the anterior lateral motor cortex (ALM) [71,72], the prefrontal cortex [73], and the primary auditory cortex (A1) [74]; note that Bitzenhofer et al. succeeded in whole-cell recordings from developing neonatal rats [73]. In addition, Guo et al. and Inagaki et al. unveiled membrane potential dynamics underlying persistent neural activity in the ALM of awake mice performing learning tasks [71,72]. More recently, Lenschow and Brecht, Ebbesen et al. and Clemens et al. investigated subthreshold membrane potential correlates of social touch of awake rodents [75,76,77]. Furthermore, several groups have reported whole-cell recordings from multiple cortical neurons of awake animals [55,57,78,79,80,81].

### 3.2. Hippocampus and Other Regions

In contrast to the neocortex, the cerebral limbic system is located inside and deeper in the brain. Regarding invasive recording from the deep region in vivo using electrodes, the probability of recording from an anticipated region per se is low because the accurate location of an electrode in the brain is blind to experimenters. In addition, for successful whole-cell recording, the tips of the patch pipettes must be kept as clean as possible to form a high-resistance seal on the cell membrane [82]. When researchers attempt to record membrane potentials from deep regions in vivo, some “obstacles” such as the extracellular matrix [83] and blood vessels [84,85] are more likely to adhere to the pipette tip. The dirty tips prevent a giga-ohm seal, causing a low success rate for whole-cell recording from the deep region of anesthetized and awake animals. Whole-cell recording from the deep regions of living animals is thus technically tough to achieve, but some researchers have attempted to resolve this issue. One of the solutions is to remove the neocortex by suction [27]. Here, we review previous studies that challenged the technical problem and provided new insights into the neural correlates of behavior [20,30,86,87,88,89,90,91].

We focus on the hippocampus and the medial entorhinal cortex, both of which are essential for the representation of the external environment. The most famous hippocampal neural correlates of the outer environment are place cells, which fire action potentials selectively when an animal crosses a specific place (called the “place field”) in the environment. While this location-specific increase in firing rates is referred to as the “rate code” of place cells, their precise spike timing relative to the phase of the ongoing hippocampal theta oscillations precedes as an animal approaches the place field (i.e., “theta phase precession”), which is called the “temporal code”. These suprathreshold activities were profiled by extracellular recordings, but intracellular dynamics for the place code remained elusive.

**Figure 4:**
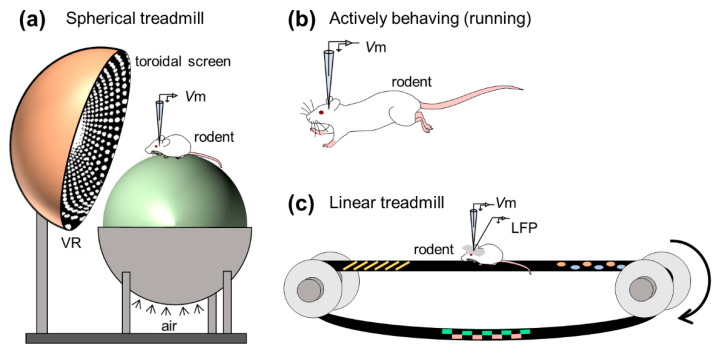
Examples of studies based on in vivo whole-cell recordings from hippocampal neurons of behaving animals. (**a**) Whole-cell recordings are made from hippocampal neurons of an animal running on the spherical treadmill with a virtual reality system. (**b**) Whole-cell recordings are made from the neurons of actively running animals. (**c**) Membrane potentials and local field potentials are simultaneously recorded from an animal running on the linear treadmill. *Abbreviations*: *V*m, membrane potential; LFP, local field potential; VR, virtual reality.

To examine the mechanisms for the dual codes, Harvey et al. first monitored the intracellular dynamics of place cells of mice navigating in a virtual-reality environment using an in vivo whole-cell recording method [86] (Figure 4a). They obtained robust location-selective firing activity of hippocampal neurons while an animal ran back and forth along a virtual linear track and identified three subthreshold signatures of place fields: (i) an asymmetric ramp-like depolarization of the baseline membrane potentials, (ii) an increase in the amplitude of intracellular theta oscillations, and (iii) a phase precession of the intracellular theta oscillations relative to the extracellularly recorded theta rhythm. These results characterized the intracellular dynamics underlying the rate and temporal codes of place cells. The virtual reality system introduced by Harvey et al. opened the door for new experimental approaches to study the neural circuits for spatial navigation. They recorded membrane potential dynamics of hippocampal place cells from head-fixed mice running on a spherical treadmill in the virtual reality system (Figure 4a).

Whole-cell recording from freely moving rodents is further technically challenging and laborious [92,93,94,95,96] (Figure 4b). This is because the active and abrupt behavior of rodents during recording causes extreme movement of the brain and often degrades a giga-ohm seal configuration. The high-resistance configuration is sensitive to even the subtle motion of brains because the diameter of a patch pipette tip is ~3 μm [97], whereas the soma of pyramidal neurons of rats and mice is approximately 20 μm in length [98,99]. Despite technical difficulties, Lee et al. first established whole-cell recordings from behaving animals [92]. Subsequently, Epsztein et al. reported fast events of membrane potentials with smaller amplitudes than spikes, which were named spikelets or fast prepotentials [87]. They subsequently examined the intrinsic differences between place cells and silent cells (i.e., cells emitting no spikes during exploration) in the hippocampal CA1 subarea while rats freely navigated the environment and found that, compared with silent cells, place cells had lower spike thresholds from the beginning of exploration and future place cells were likely to exhibit higher burst firing before exploration [100]. In another study, Lee et al. further attempted to artificially induce spatially uniform depolarization to hippocampal cells by injecting positive currents through patch pipettes and discovered that a spatially tuned subthreshold response and location-specific spiking emerged suddenly and reversibly even in silent cells [101]. Thus, they indicated that postsynaptic neuronal excitability gated presynaptic inputs and proposed a unique cellular mechanism for the generation of place codes. These studies have been followed by investigations on intracellular mechanisms for spatial representation in the rodent limbic system, including the hippocampus [102,103,104,105,106,107,108,109] and the medial entorhinal cortex [110,111].

The hippocampus generates extracellular electric oscillations, which reflect ensembles of neural suprathreshold firing and subthreshold synaptic activities and plays substantial roles in learning, memory, and spatial navigation. Characteristic extracellular oscillations (often referred to as LFPs), particularly in the hippocampus, are (i) theta oscillations (3–10 Hz) and (ii) sharp wave-ripple complexes (SWRs), consisting of sharp waves (2–30 Hz) and transient ripple oscillations (100–250 Hz), which contribute to (i) memory encoding and (ii) memory consolidation, respectively [112,113,114,115]. To seek membrane potential correlates of learning and memory, researchers have often captured subthreshold dynamics and field oscillations in the hippocampus of awake rodents, focusing especially on SWRs (but see [116,117,118] for intracellular characteristics of hippocampal neurons during other frequency bands of extracellular oscillations; Figure 4c).

English et al. first achieved intracellular recording during SWRs using sharp electrodes [119]. They succeeded in recording from freely running animals and discovered consistent large depolarizations in CA1 pyramidal cells during SWRs, which were associated with transient ripple-frequency fluctuations in the membrane potentials named intracellular ripples; note that intracellular ripples are also observed in the adjacent area (i.e., the subiculum) [120]. A series of subsequent studies precisely characterized subthreshold activity along with hippocampal SWRs [121,122,123,124]. Hulse et al. made whole-cell recordings from animals running on the treadmill and found that the membrane potentials around hippocampal ripple events consisted of (i) sharp wave-associated depolarizations, (ii) intracellular high-frequency ripple-like oscillations superimposed on extracellular ripples, and (iii) hyperpolarizations after the ripples. They further indicated that the balance between excitation and inhibition was required for precise activation of individual hippocampal pyramidal cells during SWRs [122]. These investigations on intracellular dynamics associated with SWRs suggested the synaptic mechanisms underlying the spike outputs during extracellular ripple events, which would contribute to memory consolidation.

Consistent with its contribution to memory, the hippocampus is primarily responsible for Alzheimer’s disease [125] and epilepsy [22,26]. For instance, Šišková et al. performed in vivo whole-cell recordings from hippocampal pyramidal neurons of a mouse model of Alzheimer’s disease simultaneously with high-resolution stimulated emission depletion microscopy imaging and computational modeling. They demonstrated that branching-structure-dependent amplification of synaptic inputs into action potential outputs would represent cellular pathomechanisms for network dysfunction and suggested that such pathomechanisms were potentially associated with other neurodegenerative diseases with abnormal dendritic morphology [125].

Thus far, we introduced previous studies investigating the synaptic mechanisms underlying spike outputs, especially concentrating on the neocortex, hippocampus, and hippocampal neighboring areas. In vivo whole-cell recordings from awake mammals have been further achieved in various areas, including the olfactory bulb [126], thalamus [65], cerebellum [127], lateral septum [128], and inferior colliculus of bats [129,130,131,132,133]; note that these in vivo whole-cell recording studies on the inferior colliculus were performed in awake bats because the bat inferior colliculus is not covered by either the neocortex or the cerebellum and is visually detectable through the skull.

## 4. Hybrid Methodologies with In Vivo Whole-Cell Recording Techniques

In vivo whole-cell recording in combination with other techniques not only unveils individual membrane potential dynamics but also uncovers additional characteristics of cellular activity in terms of anatomical connections, genetic properties, and collective activity associated with brain function. In this section, we introduce leading studies using such hybrid methodologies.

### 4.1. Optics

Researchers have combined in vivo whole-cell recording methods with optic techniques to record neural activity while they simultaneously (i) optogenetically manipulate specific neural activities on a restricted time scale [134,135,136,137,138,139,140,141] or (ii) capture individual cellular activity based on fluorescence [142,143].

**Figure 5:**
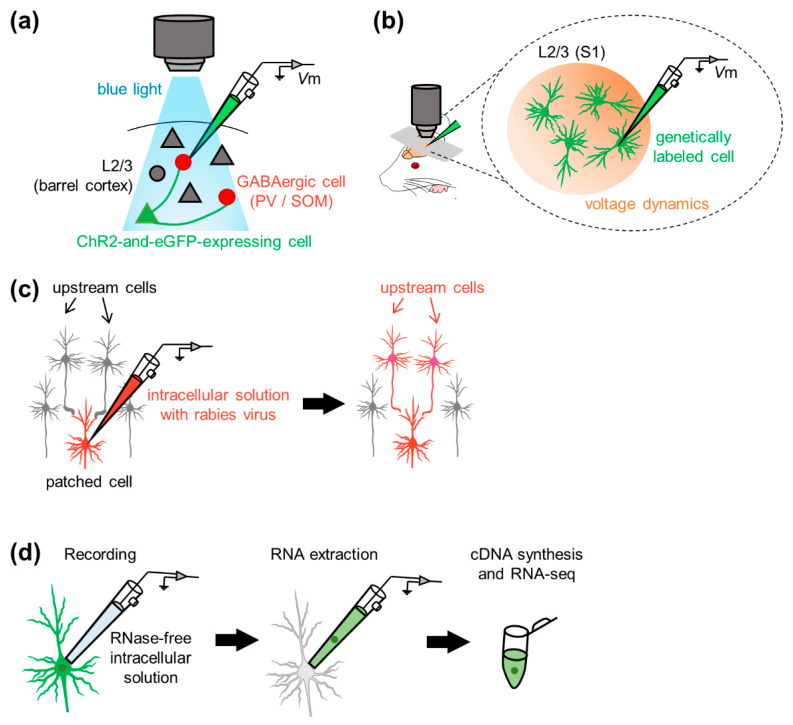
Examples of combinations of in vivo whole-cell recordings with other techniques. (**a**) Channelrhodopsin 2 and eGFP are artificially expressed in specific excitatory cells (*green*, *triangle*) in layer 2/3 of the barrel cortex. Whole-cell recordings are made from PV-positive or SOM-positive GABAergic cells (*red*, *circle*), and light-induced membrane potential fluctuations of GABAergic neurons are recorded. (**b**) Whole-cell recordings are made from neurons in layer 2/3 of the somatosensory cortex. Membrane potential fluctuations of surrounding neurons are simultaneously recorded using the voltage-sensitive dye. (**c**) Whole-cell configurations are made using the intracellular solution with the rabies virus. After recording, presynaptic (upstream) neurons are traced with the aid of the virus. (**d**) Whole-cell recordings are made using RNase-free intracellular solution. After recording, the cell content is extracted through the patch pipette. Subsequently, RNA sequencing is performed. *Abbreviations*: *V*m, membrane potential; ChR2, channelrhodopsin 2; eGFP, enhanced green fluorescent protein; PV, parvalbumin; SOM, somatostatin; S1, primary somatosensory cortex.

To optogenetically manipulate neurons, channelrhodopsins, a subfamily of retinylidene proteins (rhodopsins), are widely used [144,145,146,147,148,149]. Channelrhodopsins originally serve as sensory photoreceptors that are activated/inactivated in response to photostimulation; that is, they function as light-gated ion channels that allow ion trafficking (i.e., electric current) through the cell membrane. To combine electrophysiology with optogenetic manipulation of cellular activity in vivo; for example, researchers first genetically express channelrhodopsin-2 (ChR2) in neurons projecting to cells in the brain regions where membrane potentials are recorded. For example, Pala and Petersen delivered plasmid DNA encoding ChR2 and eGFP to an individual neuron in layer 2/3 of the mouse barrel cortex using two-photon guided electroporation and patched two subtypes of genetically labeled interneurons [136] (Figure 5a). They investigated excitatory synaptic transmission derived from individual glutamatergic excitatory neurons onto the two genetically distinct interneuron subpopulations and discovered the differences between synaptic connectivity from pyramidal cells onto two types of interneurons with respect to transmission efficacy and short-term facilitation [136]. Since they demonstrated the feasibility of empirically evaluating synaptic connectivity between specific neurons in vivo [136], neural computation and behavioral state-dependent functional connectivity between targeted neurons in the neocortical, hippocampal, and cerebellar microcircuits have been further revealed in other studies [134,138,139,141,150,151,152,153,154].

In contrast to optogenetic manipulation, either in vivo optical imaging (including voltage-sensitive dye (VSD) imaging [142,155,156,157,158,159,160] or two-photon calcium imaging [143]) was simultaneously performed with whole-cell recording to capture neural activity in a wider area than single whole-cell recording alone. The VSD imaging method requires voltage-sensitive fluorescent probes, chemical molecules that alter fluorescent intensities in response to transmembrane ionic flow (i.e., changes in transmembrane current/voltage). Petersen et al. recorded neural activity simultaneously by VSD imaging, whole-cell recording, and extracellular unit recording from layer 2/3 of rat S1 [142] (Figure 5b). They demonstrated the differences in the spatiotemporal dynamics of spontaneous and sensory-evoked activity between the two neocortical network states. This hybrid method allows for simultaneous measurement of somatic subthreshold (i.e., membrane potentials) and suprathreshold (i.e., action potentials or firing) activity dynamics and would enable us to reveal mesoscopic spatiotemporal activity patterns bridging single neuronal activity and various behavioral responses of animals.

### 4.2. Intracellular Pharmacology

Membrane potentials and currents are generated by ion channels expressed on the membrane. To unveil the contribution of specific ion channels to synaptic activity in individual neurons, researchers have pharmacologically manipulated ion conductance and attempted to reveal the contribution of excitatory and inhibitory conductance at the single-cell level [161,162]. In an early study, Nelson et al. filled patch pipettes with CsF-DIDS, a GABA_A_ receptor pore blocker, and perfused the drug into individual cells to intracellularly block chloride ion conductance and revealed that GABAergic inputs contributed to orientation selectivity in cat V1 [7]. Kobayashi et al. also used picrotoxin-filled patch pipettes to perfuse the GABA_A_ receptor antagonist into single cells and patched V1 neurons in vivo, demonstrating that GABAergic inhibition reduced the impact of some excitatory synaptic inputs on somatic excitability [163]. In contrast to the inhibitory conductance, Palmer et al. used two-photon uncaging of an intracellular NMDA receptor antagonist (tc-MK801) to locally manipulate NMDA receptors in single branches of tuft dendrites [164]. These studies with intracellular perfusion of antagonists precisely confirm the impact of specific channel conductance, whereas extracellular drug application enables us to activate/silence channel conductance in a wider range [165,166,167,168] (but see [169] for extracellular but greater local drug delivery to patched neurons).

### 4.3. Gene Manipulation: Transgene Expression and Virus-Aided Connectivity Tracing

From an anatomical point of view, while whole-cell recording enables us to determine the morphology of recorded cells with the aid of intracellular application of biocytin or neurobiotin [27,30,117,139], one of the disadvantages is that anatomical characteristics of the recorded cells and presynaptic neurons projecting to the recorded cell remain unknown. To overcome this issue, recent studies have incorporated gene manipulation techniques into whole-cell recordings. Using lentiviral vectors, Dittgen et al. delivered genes to specific neurons and analyzed the phenotypes of individual neurons [19]. Afterward, Kitamura et al. established a method of in vivo single-cell electroporation of plasmid DNA, bringing about stable transgene expression [51]. Even 24 h after electroporation, the gene-expressing neurons exhibited normal electrophysiological properties in terms of membrane potentials and action potentials.

In 2010, Marshel et al. proposed a method for labeling upstream cells of recorded neurons [170] (Figure 5c). Rancz et al. delivered DNA vectors into a given cell through patch pipettes during whole-cell recording to drive protein expression in the cell [171]. They observed stable protein expression for at least one week and found that the neurons remained intact after whole-cell recording. The gene delivery method allowed for retrograde and monosynaptic tracing of the upstream neurons that projected to patched cells in vivo [171]. With the aid of the rabies virus, Vélez-Fort et al. mapped presynaptic neurons of patched principal cells in the mouse V1. They profiled the sensory response properties and determined the monosynaptic connectivity in the cortico-cortical or cortico-thalamic loop mediating neocortical neural computations relevant to sensory perception [172]. The gene manipulation-aided identification of synaptic connectivity impinging onto the patched cell bridges the gap between the anatomical and physiological properties of neural networks [173,174,175,176,177,178,179].

### 4.4. Molecular Characterization: Patch-Seq

To link the molecular and physiological properties of single cells, researchers applied a combined method of whole-cell recording and single-cell RNA sequencing (scRNA-seq) to individual neurons in acute slices in early studies and named this method “patch-seq” [180,181,182,183,184,185,186,187]. The basic protocol of patch-seq is as follows: researchers perform whole-cell recording from individual neurons from living tissue slices or even intact animals and aspirate the cell contents through a patch pipette for subsequent RNA sequencing. Note that the morphology of the aspirated cell can be partially recovered using biocytin-filled intrapipette solution with osmolarity in a physiological range [181]. Unlike general patch-clamp recordings, glass capillaries were autoclaved prior to pulling them to obtain patch-clamp pipettes. Every surface of materials (e.g., intracellular solution) and devices (e.g., micromanipulator pieces) should be cleaned to maintain an experimental RNase-free environment during sample collection. In contrast to other scRNA-seq techniques applied to dissociated cells, patch-seq enables us to investigate single cells in situ. Cadwell et al. applied patch-seq to in vivo preparations: they advanced patch pipettes under two-photon microscopy guidance and subsequently collected RNA by applying gentle suction until the recorded cells were visually shrunken under two-photon microscopy [188] (Figure 5d). Thanks to this method, they found two types of interneurons and identified novel markers for the interneuron classes. Thus, patch-seq facilitates the characterization of neuronal subpopulations and serves as a step for the identification of undescribed neuronal subtypes in terms of the transcriptome [189].

## 5. Future Perspectives

In vivo whole-cell patch-clamp techniques have been used to observe spontaneous or sensory-evoked subthreshold dynamics in various brain regions, such as the neocortex, hippocampus, thalamus, amygdala, and others. This method was combined with optogenetic or pharmacological manipulation in not only anesthetized animals but also behaving animals during learning tasks [190]. The combination of whole-cell electrophysiology and genetic manipulation (including transgene expression and virus-mediated tracing of synaptic connectivity) is expected to bridge the gap between physiological functions and anatomical/molecular properties of individual neurons. However, these recently developed techniques have yet to be combined with whole-cell recordings from awake behaving animals, presumably because of (i) the limited number of simultaneously recorded cells and (ii) the low success rates of whole-cell configurations per se.

The VSD imaging method has been recently used to simultaneously capture multineural activities with high spatiotemporal resolution, although VSD may suffer from photobleaching [191]. In contrast to VSD, researchers have developed and improved genetically encoded voltage indicators (GEVIs) [192,193,194,195,196,197]. Although genetically encoded Ca^2+^ indicators allow us to monitor intracellular calcium transients as surrogates of neuronal electrical activity, the development of GEVIs with high voltage sensitivity and fast response kinetics makes all-optical electrophysiology (i.e., simultaneous optical perturbation and measurement of membrane potentials using light-gated channelrhodopsins and GEVIs, respectively) viable [198,199,200,201]. As GEVIs are often more restricted in the soma and more sensitive to changes in voltage than VSD, all-optical electrophysiology with GEVIs compensates for the restricted number of cells recorded at once and enables us to reveal subthreshold membrane potential correlations/oscillations/dynamics. Moreover, cell-type-specific classification and characterization of neuronal activities are feasible when GEVIs are knocked in at some loci [199]. However, strictly speaking, all-optical electrophysiology does not allow us to precisely record absolute values of membrane potentials and to describe the intrinsic excitability of neurons. Moreover, unlike the whole-cell recording method, all-optical electrophysiology is unable to monitor excitatory/inhibitory postsynaptic currents by clamping the voltage or to profile intrinsic properties (such as membrane capacitance/resistance/time constant) by injecting depolarizing currents into cells. Sag potentials in response to hyperpolarizing currents [202] may not be monitored in all-optical electrophysiology. Furthermore, all-optical electrophysiology is not suitable for single-cell pharmacological manipulation (as already discussed in Section 4.2). Thus, when a precise (i.e., high signal-to-noise ratio) or pharmacological (e.g., channel dependency) description of subthreshold dynamics is required, the whole-cell recording method should be the first choice among a variety of methods for recording neuronal activity.

The biggest issue of the whole-cell recording method might be its low success rate and the small number of simultaneously accessible cells. One of the solutions can be the automation of the process [203,204,205,206,207,208,209,210]. Several groups have developed patch-clamping robots and succeeded in obtaining in vivo whole-cell recordings from the neocortex and the hippocampus [203,206,211], even from deeper areas (e.g., the thalamus) [212]. Recently, Annecchino et al. and Suk et al. established automation of in vivo targeted patch-clamp recording [204,208]. When combined with anterograde tracing, this automated method may help researchers record the membrane potentials of multiple targeted cells innervated by specific neurons. Moreover, multiple pairwise comparisons of subthreshold dynamics between excitatory neurons and various types of targeted interneurons would be fascinating. Surprisingly, more recently, Kodandaramaiah et al. developed patching robots enabling whole-cell recordings simultaneously from up to four neurons [205]. However, even automated patching methods do not allow recordings from as many cells as extracellular unit recordings or optical imaging techniques. In that regard, we could partly overcome the problem if we combined the whole-cell patch-clamp method with other techniques that enable us to capture multineural activities. For example, simultaneous recordings of membrane potentials from neurons in a given region and extracellular firing activities in the upstream region would lead to more precise characterization of individual neuronal properties among functionally identified cell assemblies. These combined methodologies sublimate the existing knowledge to unified comprehension of the neural circuitry supporting behavioral functions.

Recently, in vivo whole-cell recordings have been applied in not only rodents but also other various species of living animals, including zebrafish [213,214] (see [214] for the ex vivo condition), ferrets [215], cats [5,216,217,218] (see [218] for recording from glial cells), bats [129], nonhuman primates (marmosets [53] and macaque monkeys [69]), and even nematodes (*Caenorhabditis elegans*) [219]. Moreover, researchers have succeeded in in vitro patch-clamp recordings from dendrites [220] and axons [221] of neurons in slice preparations. Furthermore, in vivo dendritic patch-clamp recordings have been reported recently [51]. The application of axonic patching to in vivo preparations may reveal novel computational mechanisms enabling the diverse behavior of animals. Thus, future in vivo whole-cell recordings would shed light upon new physiological underpinnings that link various brain functions to anatomical, molecular, and genetic characteristics.

## Data Availability

Not applicable.
